# Outcomes of permanent canines on the cleft side after secondary alveolar grafting using different materials in complete unilateral cleft lip and palate

**DOI:** 10.1590/1678-7757-2022-0478

**Published:** 2023-05-01

**Authors:** Bruno Mariano Ribeiro Braga, Claudia Resende Leal, Roberta Martinelli Carvalho, Gisele da Silva Dalben, Terumi Okada Ozawa

**Affiliations:** 1 Universidade de São Paulo Hospital de Reabilitação de Anomalias Craniofaciais Programa de Pós Graduação em Ciências da Reabilitação São Paulo Bauru Brasil Universidade de São Paulo, Hospital de Reabilitação de Anomalias Craniofaciais, Programa de Pós Graduação em Ciências da Reabilitação, São Paulo, Bauru, Brasil.; 2 Universidade de São Paulo Hospital de Reabilitação de Anomalias Craniofaciais Departamento de Cirurgia Bucomaxilofacial São Paulo Bauru Brasil Universidade de São Paulo, Hospital de Reabilitação de Anomalias Craniofaciais, Departamento de Cirurgia Bucomaxilofacial, São Paulo, Bauru, Brasil.; 3 Universidade de São Paulo Hospital de Reabilitação de Anomalias Craniofaciais Departamento de Odontopediatria São Paulo Bauru Brasil Universidade de São Paulo, Hospital de Reabilitação de Anomalias Craniofaciais, Departamento de Odontopediatria, São Paulo, Bauru, Brasil.; 4 Universidade de São Paulo Hospital de Reabilitação de Anomalias Craniofaciais Departamento de Ortodontia e Ortopedia Facial São Paulo Bauru Brasil Universidade de São Paulo, Hospital de Reabilitação de Anomalias Craniofaciais, Departamento de Ortodontia e Ortopedia Facial, São Paulo, Bauru, Brasil.

**Keywords:** Cleft lip, Cleft palate, Bone transplantation

## Abstract

The prevalence of impaction of the permanent canine on the cleft side (PCCS) ranges from 12-35% after alveolar bone grafting (ABG). PCCSs usually develop above other permanent teeth in the alveolar process, gradually becoming vertical until they reach the occlusal plane. The type of cleft, hypodontia of lateral incisor on the cleft side, slower PCCS root development, and genetic factors are predictors of impaction and/or its ectopic eruption.

**Objective:**

To compare the behavior of PCCS in individuals with complete unilateral cleft lip and palate (UCLP) subjected to secondary alveolar grafting (SAG) with different materials.

**Methodology:**

This retrospective longitudinal study analyzed 120 individuals undergoing SAG with iliac crest bone, rhBMP-2, and mandibular symphysis. The individuals were selected at a single center and equally divided into three groups. Panoramic radiographs were analyzed by the Dolphin Imaging 11.95 software to measure PCCS angulation and PCCS height from the occlusal plane at two different timepoints.

**Results:**

No statistical significance was found between grafting materials (P=0.416). At T1, the PCCS height from the occlusal plane was greater for rhBMP-2 and mandibular symphysis compared to iliac crest bone. The lateral incisor on the cleft side was not related to success or lack of eruption of PCCS (P=0.870).

**Conclusion:**

Impaction rates of PCCS were similar for the materials studied. Absence of the lateral incisor on the cleft side did not prevent spontaneous eruption of PCCSs.

## Introduction

The prevalence of permanent canine impaction on the cleft side ranges from 12-35% after secondary alveolar bone grafting (ABG)^1,2^ compared to 1.7-3% in the general population.^3,4^ Maxillary canines usually develop above other permanent teeth in the alveolar process. During spontaneous eruption, the canine moves toward the occlusal plane and gradually becomes vertical. Permanent canine on the cleft side (PCCS) erupts more slowly, with delayed root development compared to the contralateral side. This long-lasting eruption makes it more susceptible to ectopia and increases the risk of impaction.^5,6^ Furthermore, a relationship between tooth agenesis, clefts, and genetic disorders has been suggested.^5,7^ Studies have shown that an increased mesiodistal angulation and a more apical position of PCCS are associated with its impaction.^2,5,8-10^ Some authors have associated impacted canines with the lack of bone in alveolar defects, which reduces the space available for eruption.^9^ Also, timing of ABG surgery can also interfere with PCCS eruption.^5,11^

Secondary alveolar bone grafting (SABG) is performed at mixed dentition, preferably before PCCS eruption, due to its contribution to maintenance of the grafted site and neighboring supporting periodontium.^12–14^ Besides favoring spontaneous eruption of PCCS, the main advantage of SABG is to allow orthodontic movement into the grafted alveolar cleft without harming periodontal tissues. Other benefits of SABG include stabilization of maxillary segments and closure of oronasal fistulas.^15^ Autogenous marrow and cancellous bone from the iliac crest (IC) are considered the gold standard for SABG due to their non-immunogenicity, osteogenic, osteoinductive and osteoconductive capacity, and greater material supply capacity.^16,17^ However, bone harvesting from the IC results in some morbidity and unsightly scarring. Thus, studies have been conducted on alternative materials to IC.

SABGs using recombinant human bone morphogenetic protein (rhBMP-2) in absorbable collagen sponge^19^ or mandibular symphysis (MS)^18^ have shown similar outcomes when compared with those from IC. Furthermore, operating exclusively intraorally has been experienced as a less extensive surgery by patients and their parents compared to using the IC as a donor site.^18^ Prevalence of canine impaction in patients with CLP after ABG using IC was 20 times higher compared to canine impaction in non-cleft individuals.^5^ However, studies comparing both MS and IC^1^ and rhBMP-2 and IC^20^ have shown similar impaction rates between grafting materials.

The prevalence of canine impaction on the cleft side and its possible risk factors have been widely reported in the literature. Although this higher prevalence has not been completely elucidated, it is probably related to both local tooth and cleft site factors. Thus, this study aimed to compare the canine eruption on the cleft side grafted with IC, MS, or rhBMP-2. The null hypothesis was that canine impaction would be similar between these different types of materials and that other local factors would be associated with tooth impaction on the cleft side.

## Methodology

This longitudinal retrospective study was approved by the Institutional Review Board of the Hospital for Rehabilitation of Craniofacial Anomalies of the University of São Paulo (HRAC-USP) (CAAE: 51860021.2.0000.544; protocol no. 5.072.476) and was conducted in accordance with the Declaration of Helsinki. All patients received and signed an informed consent form. The sample size estimated considered an 80% statistical power with a 5% alpha error, a 14.1°^2^ standard deviation and a 10° minimum intergroup difference. A minimum sample size of 37 patients was required for each group.

In total, 120 individuals (85 males and 35 females) were selected at a single center from 2006 to 2020. Patients were consecutively selected according to AG material and divided equally into three groups. Inclusion criteria were complete unilateral cleft lip and palate, and successful SAG surgery performed before PCCS eruption based on the Bergland scale modified by Williams, et al.^21^ (2003). All surgeries were performed by the maxillofacial team of HRAC-USP using iliac crest bone, rhBMP-2, or mandibular symphysis bone. Anatomical cleft characteristics and institutional availability were considered to determine the grafted material. This information was collected from medical records. Exclusion criteria were associated craniofacial syndromes, comorbidities, SAG failure, and absence of preoperative or postoperative SAG radiographs. Mean age of groups was 11 years and four months (SD±11 months) for iliac crest group (ICG), 10 years and four months (SD±12 months) for rhBMP-2 group (BMPG), and 10 years and 10 months (SD±13 months) for mandibular symphysis group (MSG).

**Figure 1:**
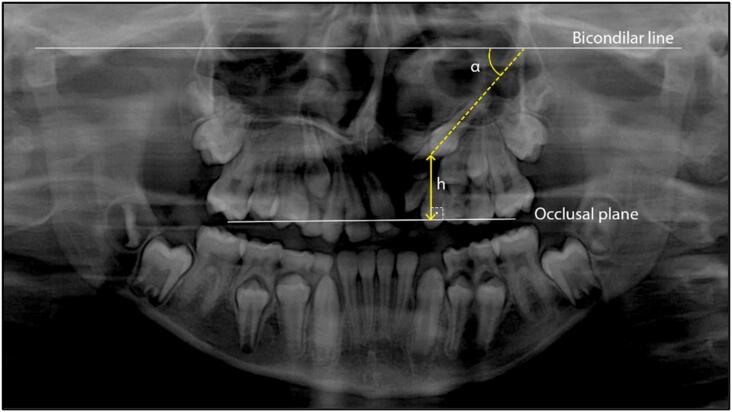
A bicondylar line superior to the condyles was drawn and used as reference to measure the mesiodistal angulation of the permanent canine on the cleft side (α).^21–23^ As α decreased, the canine mesiodistal angulation increased. The height of the permanent canines on the cleft side was measured in relation to the occlusal plane by the line drawn from the tip of the mesiobuccal cusp of the maxillary first permanent molars (h).^21–23^

The position of the permanent canine on the cleft side was analyzed using panoramic radiographs. The panoramic radiographs were taken one month (SD±3 months) before (T1) and 15 months (SD±8 months) after SAG surgery (T2). Mesiodistal angulation of PCCS was evaluated by the angle between the long axis of PCCS and the drawn bicondylar line.^20,22,23^ PCCS height was measured from its cusp tip to the occlusal plane, perpendicularly (Figure 1),^22,23^ using the Dolphin Imaging (11.95) Software (Patterson Dental Supply, Inc., Chatsworth, California, USA) (Table 1).^20^

**Table 1:** Sample general characteristics

Group	PCCS eruption		Mean (SD) at T1				Mean (SD) at T2			
	Erupted	Non-erupted	Erupted		Non-erupted		Erupted		Non-erupted	
			Angulation (°)	Height (mm)	Angulation (°)	Height (mm)	Angulation (°)	Height (mm)	Angulation (°)	Height (mm)
ICG	28	12	64.0 (±9.7)	4.3 (±2.4)	61.6 (±13.47)	7.2 (±1.9)	77.3 (±9.2)	-2.2 (±2.4)	70.0 (±13.1)	3.1 (±3.1)
BMPG	32	8	65.9 (±10.8)	9.3 (± 4.5)	54.1 (±13.0)	11.9 (±2.9)	72.9 (±11.7)	1.2 (±5.3)	68.2 (±15.2)	0.5 (±4.4)
MSG	27	13	68.3 (±9.1)	7.5 (±3.4)	53.1 (±9.0)	13.5 (±3.2)	76.5 (±9.3)	-0.9 (±3.7)	63.2 (±20.4)	4.6 (±5.7)

PPCS, permanent canine on the cleft side; SD, standard deviation; ICG, iliac crest group; BMPG, rhBMP-2 group; MSG, mandibular symphysis group.

Moreover, agenesis of the cleft lateral incisor and development of PCCS at T1 were considered. PCCS root calcification stage was based on Nolla’s classification.^24^

### Statistics analysis

Panoramic radiographs were analyzed by two blinded and independent examiners. Measurements were repeated with a 7-day interval for assessment of intra-examiner reliability. Method error was determined using Intraclass Correlation Coefficient (ICC). Systematic and random errors were evaluated using *t*-test and Dahlberg’s formula, respectively. Interphase and intergroup comparisons were performed using analysis of variance and the chi-square test (p<0.05). The software used for statistical analysis was SPSS Statistical (IBM Corporation, New York, USA).

## Results

Intra-examiner reliability for PCCS angulation and PCCS height measurements was excellent, with ICCs=0.996 and 0.994, respectively. ICCs for inter-examiner reliability were also excellent (0.963 and 0.992), as shown in Table 2. Intra-examiner and inter-examiner random error for PCCS angulation ranged from 1.17-3.55°, respectively. For PCCS height, intra-examiner and inter-examiner random error ranged from 0.63-0.74 mm, respectively. Only PCCS angulation showed a significant inter-examiner systematic error (P=0.007) (Table 2).

**Table 2:** Intra- and Inter-examiner reliability for angulation and height of PCCS (ICC, Dahlberg’s Formula, and *t*-test)

	Angulation			Distance		
	ICC, Dahlberg's Formula, and t-test			ICC, Dahlberg's Formula, and t-test		
Intra-examiner reliability	0.996	1.17°	P=0.488	0.994	0.63 mm	P=0.466
Inter-examiner reliability	0.963	3.55°	P=0.007*	0.992	0.74 mm	P=0.060

* statistical significance.

ICC, intraclass correlation coefficient; PCCS, permanent canine on the cleft side.

**Figure 2:**
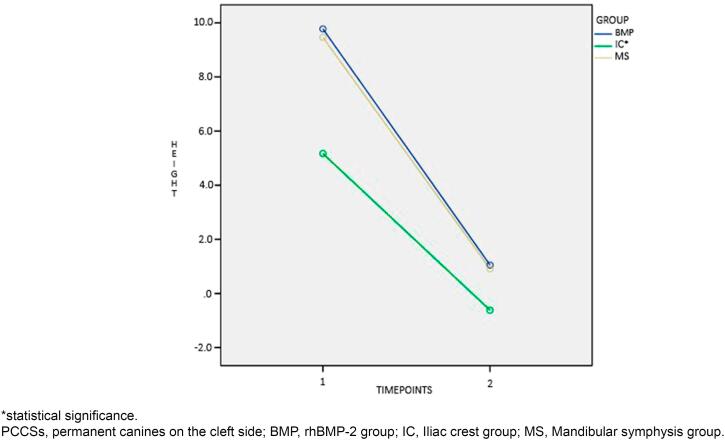
Interaction of time for PCCSs height (mm) from the occlusal plane

**Figure 3:**
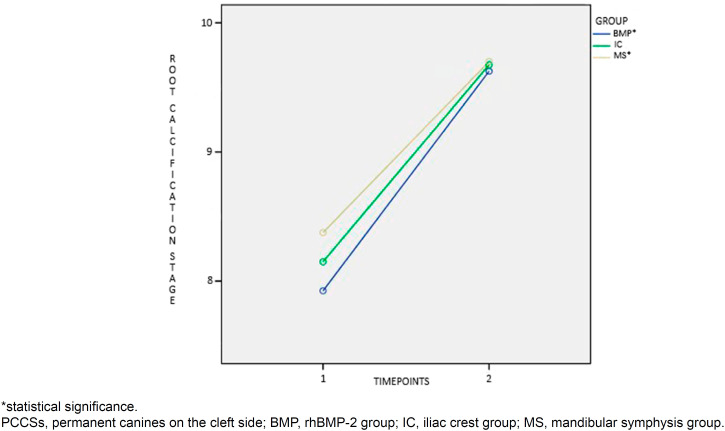
Interaction of time for PCCSs root calcification stage

**Figure 4:**
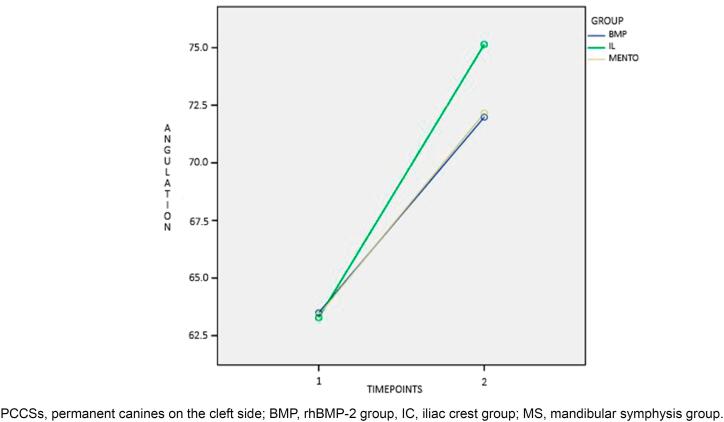
Interaction of time for PCCSs angulation(°)

Before SAG, differences between groups were found for PCCS height (Figure 2), root formation stage (Figure 3), and mean age (P<0.001). Patients in the ICG were on average one year and one month older than patients in the BMPG. In total, 87 (72.5%) PCCS erupted spontaneously, without statistically significant difference between materials used for the reconstruction of residual alveolar defects (P=0.416). Interaction of time was found for PCCS angulation (P<0.001, Figure 4), PCCS height (P<0.001, Figure 2), and root formation stage (P<0.001, Figure 3). PCCS height showed a group and time interaction (P=0.002, Figure 2) and a group effect (P<0.001, Figure 2). A significant difference was observed between ICG against BMPG (P<0.001) and ICG against MSG (P<0.001) at T1: PCCSs were higher by 4.6 1mm and 4.29 mm in BMPG and MSG, respectively. Individuals in the MSG presented stage 9 of root formation more frequently compared to those in the BMPG (P<0.040) and ICG (P=0.511), who presented predominance of stage 8.

Intragroup comparison was significant for PCCS angulation, PCCS height, and root formation stage between T1 and T2 (P<0.001, Figures 2, 3, and 4). The cleft lateral incisor was not statistically related to the success or failure of PCCS eruption (P=0.870). In total, 81 (72.3%) PCCS erupted spontaneously without a cleft lateral incisor as a guide. Of 81 canines, 34.6% belonged to the ICG, 37% to the BMPG, and 28.4% to the MSG.

## Discussion

The null hypothesis that the SAG material does not influence PCCS outcome was confirmed (P=0.416) and agrees with previous studies that showed similar rates of canine eruption into the cleft site grafted by iliac crest, rhBMP-2, or mandibular symphysis.^5,17^ The panoramic radiograph is used to determine the angulation and height of canines^4,18,20,21,23^ and has shown reproducibility and reliability of the technique in previous studies.^18,20,23^ Similarly, in this study, intra-examiner and inter-examiner reliabilities for angulation and height ranged from 0.96-0.99 ICC. Intra-examiner and inter-examiner random error for PCCS angulation was less than 4°. Intra-examiner and inter-examiner random error for PCCS height was less than 1.00 mm (Table 2). Although PCCS angulation showed a significant systematic error (P=0.007, Table 2), the quality of some older panoramic radiographs impaired the identification of condyles. A previous study also reported difficulty in identifying the condyle landmark on some panoramic radiographs,^25^ which encourages the use of radiographs with better quality and does not make the method unfeasible.

Despite the attempt to pair groups in starting forms to eliminate biases, differences between groups were found for PCCS height, root formation stage, and mean age at T1. Although patients in the ICG were chronologically older than those in the BMPG, with no statistically significant difference between the chronological ages of ICG and MSG, tooth development was more delayed in both ICG and BMPG compared to MSG (Figure 3). This probably occurred because bone harvesting from the mandibular symphysis is safer when mandibular permanent canines have more developed roots.^18,26–28^

The similarity of the three graft materials compared in this study concerning the ability to fill the cleft was previously shown.^18,19,29,30^ MS was an attractive donor site with low morbidity compared to IC. Its advantages include restriction to intraoral operative sites, minimal pain or discomfort, and an invisible scar in the lower labial sulcus.^1,26^ The restriction to the intraoral operative site was also a disadvantage since it does not allow two teams to operate simultaneously, leading to longer operations. Other disadvantages of the donor site include limited supply, making it not suitable for large or bilateral clefts,^18,31^ impairment of neighboring teeth and mental nerve, and an increased rate of impacted canines.^1,26,27^

In this study, the ratio of impacted canines at grafted sites that required surgical exposure for orthodontic traction was similar to the previous literature (27.5% against 12-35%).^5,8^ Of 33 unerupted canines, 28 underwent surgical exposure and orthodontic traction. Five canines are being followed and will likely require surgical exposure for orthodontic traction. Of 28 orthodontically tractioned canines, 12 were in the ICG, eight in the BMPG, and eight in the SMG.

In agreement with previous studies that showed that late alveolar bone grafting may be associated with an increased canine angulation and a higher canine position in the horizontal and vertical sectors,^5,11^ a time interaction was found in intergroup and intragroup comparisons for PCCS angulation, PCCS height, and root formation stage in this study. An increased mesiodistal angulation and greater height were also observed as predictors of canine impaction, confirming a previous study.^20^ In our study, impacted canines showed mean differences of approximately 11° and 8mm in angulation and height before and after AG, respectively. Although AGs were performed before PCCS eruption, starting differences in root development may have influenced PCCS behavior. Future studies should compare PCCS outcomes after AG performed at the same root formation stage. Also, a study mentioning more details about the final position of PCCS after AG should be carried out. Panoramic radiographs analyzed varied in timing standardization. Possible explanations would be that the institution is a teaching hospital and serves patients with socioeconomic difficulties, making protocol follow-ups unfeasible.

## Conclusion

Impaction rates of permanent canines on the cleft side were similar between ABG performed with iliac crest, rhBMP-2, and mandibular symphysis. The absence of the lateral incisor on the cleft side did not prevent spontaneous eruption of PCCS.
